# Supplementary material for: A Computational Framework for Comprehensive Genomic Profiling in Solid Cancers: The Analytical Performance of a High-Throughput Assay for Small and Copy Number Variants
Source: Cancers (Basel). 2022 Dec 13;14(24):6152. doi: 10.3390/cancers14246152 (PMC9776229; doi:10.3390/cancers14246152)
Supplement: Supplementary file 1 [file cancers-14-06152-s001.zip › cancers-2023731-supplementary.docx]

**Supplementary Materials**

**Supplementary Table S1**. Annotation and comparison of expected and observed DNA variants. Table attributes are defined as follows: **Disease**, tissue from which the sample was taken; **Gene**, investigated gene from previous detection method; **Sample**, patient sample ID; **Run**, sequencing run ID; **Exon** **median** **coverage**, median depth of coverage in the investigated genes; **Expected** **alteration**, Alteration detected by the previous method; **Previous** **detection** **method**, methodology used before the TSO500 assay; **Alteration** **type**, Type of alteration detected by the previous methodology; **Detected**, if the previous alteration is identified by the TSO500 assay; **VAF** **(%) expected**, variant allele frequency expected from the previous method; **VAF (%)** **detected**, variant allele frequency detected by the TSO500 assay. CNV keys: tCNV pos, somatic CNV positive test; gCNV pos, germline CNV positive test; CNV neg, CNV negative test.

| **Disease** | **Gene** | **Sample** | **Run** | **Exon median coverage (u)** | **Expected**  **Alteration** | **Previous detection Method** | **Alteration Type** | **Detected** | **VAF(%) Expected**^§^ | **VAF(%)**  **Detected**^§^ |
| --- | --- | --- | --- | --- | --- | --- | --- | --- | --- | --- |
| Breast | BRCA1 | TSO500_D001 | 2 | 865 | c.5035_5039del, p.Leu1679YfsTer2 | NGS | Frameshift | Yes | 69 | 72 |
|  | PIK3CA | TSO500_D002 | 5 | 436 | c.3140A>G, p.His1047Arg | Sanger | Missense | Yes |  |  |
|  | PIK3CA | TSO500_D003 | 5 | 520 | c.3140A>G, p.His1047Arg | Sanger | Missense | Yes |  |  |
|  | ERBB2 | TSO500_D004 | 5 | 656 | CNV 3+ | FISH | CNV | Yes |  |  |
| Colon | KRAS | TSO500_D005 | 1 | 1152 | c.34G>C^34G>A^35G>T^35G>A^35G>C, p.Gly12Xaa | real-time PCR | Missense | Yes |  |  |
|  | KRAS | TSO500_D006 | 1 | 1283 | c.34G>T, p.Gly12Cys | real-time PCR | Missense | Yes |  |  |
|  | KRAS | TSO500_D007 | 2 | 792 | c.38G>A, p.Gly13Asp | real-time PCR | Missense | Yes |  |  |
|  | NRAS | TSO500_D008 | 2 | 833 | c.34G>A^34G>T^35G>C^35G>A^35G>T^37G>C^38G>A^38G>T, p.Gly12Xaa-Gly13Xaa | real-time PCR | Missense | Yes |  |  |
|  | NRAS | TSO500_D009 | 2 | 435 | c.181C>A, p.Glu61Lys | real-time PCR | Missense | Yes |  |  |
|  | BRAF | TSO500_D010 | 2 | 1118 | c.1799T>A, p.Val600Glu | real-time PCR | Missense | Yes |  |  |
|  | KRAS | TSO500_D011 | 4 | 119 | c.436G>A^436G>C^437C>T, p.Ala146Xaa | real-time PCR | Missense | Yes |  |  |
|  | KRAS | TSO500_D012 | 4 | 314 | c.34G>C^34G>A^35G>T^35G>A^35G>C, p.Gly12Xaa | real-time PCR | Missense | Yes |  |  |
|  | KRAS | TSO500_D013 | 4 | 250 | c.34G>C^34G>A^35G>T^35G>A^35G>C, p.Gly12Xaa | real-time PCR | Missense | Yes |  |  |
|  | KRAS | TSO500_D014 | 4 | 705 | c.34G>C^34G>A^35G>T^35G>A^35G>C, p.Gly12Xaa | real-time PCR | Missense | Yes |  |  |
|  | KRAS | TSO500_D015 | 4 | 239 | c.34G>C^34G>A^35G>T^35G>A^35G>C, p.Gly12Xaa | real-time PCR | Missense | Yes |  |  |
|  | KRAS | TSO500_D016 | 4 | 289 | c.436G>A^436G>C^437C>T, p.Ala146Xaa | real-time PCR | Missense | Yes |  |  |
|  | KRAS | TSO500_D017 | 4 | 380 | c.34G>C^34G>A^35G>T^35G>A^35G>C, p.Gly12Xaa | real-time PCR | Missense | Yes |  |  |
|  | KRAS | TSO500_D018 | 4 | 112 | c.34G>C^34G>A^35G>T^35G>A^35G>C, p.Gly12Xaa | real-time PCR | Missense | Yes |  |  |
|  | KRAS | TSO500_D019 | 5 | 480 | c.34G>T, p.Gly12Cys | real-time PCR | Missense | Yes |  |  |
|  | KRAS | TSO500_D020 | 5 | 709 | c.35G>T, p.Gly12Val | real-time PCR | Missense | Yes |  |  |
|  | KRAS | TSO500_D021 | 5 | 166 | c.35G>A, p.Gly12Asp | real-time PCR | Missense | Yes |  |  |
|  | KRAS | TSO500_D022 | 5 | 590 | c.34G>C^34G>A^35G>T^35G>A^35G>C, p.Gly12Xaa | real-time PCR | Missense | Yes |  |  |
| GIST | Ckit | TSO500_D023 | 1 | 1215 | c.1661_1675del, p.Glu554_Lys558del | Sanger | indel | Yes |  |  |
|  | Ckit | TSO500_D024 | 3 | 1083 | c.1670_1684delinsACAC, p.Trp557_Glu562delinsTyrThr | Sanger | indel | Yes |  |  |
|  | PDGFRA | TSO500_D025 | 5 | 524 | c.1750_1757delinsCT, p.Ser584_Trp586delinsLeu | Sanger | indel | Yes |  |  |
|  | PDGFRA | TSO500_D026 | 5 | 709 | c.1671_1676del p.Trp557_Val559delinsCys | Sanger | indel | Yes |  |  |
|  | PDGFRA | TSO500_D027 | 5 | 837 | c.2527_2541delinsTGTCTC p.Ile843_SerdelinsCysLeu | Sanger | indel | Yes |  |  |
|  | Ckit | TSO500_D028 | 5 | 265 | c.1669T>A p.Trp557Arg | Sanger | Missense | Yes |  |  |
| Lung | KRAS | TSO500_D029 | 1 | 1306 | c.35G>A, p.Gly12Asp | Sanger | Missense | Yes |  |  |
|  | EGFR | TSO500_D030 | 3 | 1158 | c.2155G>A^2155G>T^2156G>C^2156G>-, p.Gly719Xaa | real-time PCR | Missense | Yes |  |  |
|  | EGFR | TSO500_D031 | 3 | 394 | c.2573T>G, p.Leu858Arg and c.2369C>T, p.Thr790Met | real-time PCR | Missense | Yes |  |  |
|  | EGFR | TSO500_D032 | 5 | 535 | del19 | real-time PCR | indel | Yes |  |  |
|  | EGFR | TSO500_D033 | 5 | 1051 | c.2573T>G, p.Leu858Arg | real-time PCR | Missense | Yes |  |  |
|  | EGFR | TSO500_D034 | 5 | 676 | ins20 | real-time PCR | indel | Yes |  |  |
| Melanoma | BRAF | TSO500_D035 | 5 | 777 | c.1799T>A, p.Val600Glu | real-time PCR | Missense | Yes |  |  |
| Ovary | DICER1 | TSO500_D036 | 1 | 786 | DICER1c.5438A>G, p.E1813G | NGS (CNV)/Sanger(Missense) | Missense | Yes |  |  |
|  | BRCA1 | TSO500_D037 | 1 | 457 | c.5503C>T, p.(Arg1835Ter) + CNV somatic BRCA1 (ex1, ex8) | NGS | Nonsense CNV | CNV not detected | 73 | 71 |
|  | BRCA1 / TP53 / NF1 / PIK3CA / FGF6 / MSH6 / TMB / MSI | TSO500_D038 | 1 | 743 | BRCA1 CNV(dup ex7) / TP53 CNV(dup ex1-9) / NF1 c.6981_5988delCAAAAATA p.Arg1994frameshiftTer12 / PIK3CA c.2702G>T p.Cys901Phe / FGF6 c.594C>G p.Ile198.Met / MSH6 CNV (del ex4) / TMB 6 Mut/Mb / MSI stable | NGS | CNV / frameshift/ missense | CNVs not detected |  |  |
|  | MSH2 | TSO500_D039 | 1 | 1646 | c.294T>G, p.Tyr98Ter | NGS/Sanger | Missense | Yes | 50 | 53 |
|  | BRCA2 | TSO500_D040 | 2 | 700 | c.5701G>T, p.Glu1901Ter | NGS | Missense | Yes | 27 | 40 |
|  | BRCA1 | TSO500_D041 | 2 | 537 | c.718C>T, p.Gln240Ter | NGS | Missense | Yes | 17 | 25 |
|  | BRCA1/2 | TSO500_D042 | 3 | 778 | BRCA2 c.3358G>T, p.Glu1120Ter / BRCA1-2 CNVneg | NGS | Nonsense / CNV | Yes | 8 | 53 |
|  | BRCA1 | TSO500_D043 | 3 | 1127 | c.4964_4982del, p.Ser1655TyrfsTer16 | NGS | Frameshift | Yes | 69 | 76 |
|  | BRCA2 | TSO500_D044 | 3 | 817 | c.1543G>T, p.Glu515Ter | NGS | Missense | Yes | 63 | 63 |
|  | BRCA1/2 | TSO500_D045 | 3 | 686 | c.3643G>A, p.Gly1215Arg / BRCA-CNVneg | NGS | Missense / CNV | Yes | 58 | 58 |
|  | BRCA1/2 | TSO500_D046 | 3 | 843 | BRCA2 c.3326del, p.Ala1109GlufsTer10/ BRCA1-2 CNVneg | NGS | Fameshift / CNV | Yes | 63 | 62 |
|  | BRCA1/2 | TSO500_D047 | 4 | 403 | BRCA2 c.10252A>C, p.Ile3418Leu, BRCA1-2 CNVneg | NGS | Missense / CNV | Yes | 59 | 59 |
|  | BRCA1/2 | TSO500_D048 | 4 | 202 | BRCA2 c.6131G>C, p.Gly2044Ala, BRCA1-2 CNVneg | NGS | Missense / CNV | Yes | 49 | 46 |
|  | BRCA1 | TSO500_D049 | 4 | 598 | c.172C>A, p.Pro58Thr | NGS | Missense | Yes | 64 | 64 |
|  | BRCA1 | TSO500_D050 | 4 | 217 | c.215G>T, p.Ser72Ile | NGS | Missense | Yes | 8 | 11 |
|  | BRCA2 | TSO500_D051 | 4 | 99 | c..215A>G, p.Asn72Ser | NGS | Missense | Yes | 79 | 77 |
|  | BRCA1 | TSO500_D052 | 4 | 473 | c.787+1G>T, p.Gly263Val | NGS | Missense | Yes | 35 | 24 |
|  | BRCA1/2 | TSO500_D053 | 4 | 1137 | BRCA neg, CNV neg | NGS | - | Yes |  |  |
|  | MTYH | TSO500_D054 | 4 | 713 | MTYH c.64G>A, p.Val22Met | NGS | Missense | Yes | 57 | 60 |
|  | BRCA1 | TSO500_D055 | 5 | 718 | gCNV pos | NGS/MLPA | CNV | CNV not detected |  |  |
|  | BRCA1 CNV | TSO500_D056 | 5 | 689 | gCNV pos | NGS/MLPA | CNV | CNV not detected |  |  |
|  | BRCA2 CNV | TSO500_D057 | 5 | 1363 | c.5796_5797delTA, p.His1932GlnfsTer12, gCNV pos | NGS/MLPA | Frameshift / CNV | CNV not detected | 55 | 48 |
|  | BRCA1 CNV | TSO500_D058 | 5 | 543 | tCNV pos | NGS/MLPA | CNV | CNV not detected |  |  |
|  | BRCA1 CNV | TSO500_D059 | 5 | 486 | gCNV pos | NGS/MLPA | CNV | CNV not detected |  |  |
|  | BRCA 2 CNV | TSO500_D060 | 5 | 266 | tCNV pos | NGS/MLPA | CNV | CNV not detected |  |  |
|  | BRCA1 | TSO500_D061 | 5 | 815 | gCNV pos | NGS/MLPA | CNV | CNV not detected |  |  |
|  | BRCA1 CNV | TSO500_D062 | 5 | 260 | gCNV pos | NGS/MLPA | CNV | CNV not detected |  |  |
|  | BRCA1 CNV | TSO500_D063 | 5 | 547 | gCNV pos | NGS/MLPA | CNV | CNV not detected |  |  |
|  | BRCA1 CNV | TSO500_D064 | 5 | 652 | gCNV pos | NGS/MLPA | CNV | CNV not detected |  |  |
|  | BRCA1/2 | TSO500_D065 | 5 | 892 | BRCA1 c.2671dup, p.Ser891PhefsTer12 /BRCA2 c.6065C>T, p.(Ser2022Leu) | NGS | Frameshift Missense | Yes | 32-31 | 44-41 |
|  | BRCA1 CNV | TSO500_D066 | 5 | 245 | gCNV pos | NGS/MLPA | CNV | CNV not detected |  |  |
| Pancreas | BRCA1/2 | TSO500_D067 | 5 | 899 | BRCA neg, CNV neg | NGS | - | Yes |  |  |
| Prostate | BRCA 2 | TSO500_D068 | 2 | 909 | c.2808_2811del, p.Ala938ProfsTer21 / c.36dup, p.Glu13Ter / BRCA2 del ex12-27 | NGS/MLPA | Frameshift Nonsense CNV | Dup and CNV not detected | 44-39 | 44-3 |
| Thyroid | RET | TSO500_D069 | 5 | 471 | RET- | Sanger | - | Yes |  |  |
|  | RET | TSO500_D070 | 5 | 803 | c.2410G>A, p.Val804Met | Sanger | Missense | Yes |  |  |
| Control | Horizon | TSO500_D071 | 5 | 727 | reference | NGS | ref | Yes | ref | ref |

§: available only for NGS methodology.

**Supplementary Table S2**. Wet quality metrics.

|  | **Overall** | | | **Run 1** | | | **Run 2** | | | **Run 3** | | | | | **Run 4** | | | **Run 5** | | |  |
| --- | --- | --- | --- | --- | --- | --- | --- | --- | --- | --- | --- | --- | --- | --- | --- | --- | --- | --- | --- | --- | --- |
| LP (Manual/Hamilton/Hybrid) |  | | | Manual | | | Manual | | | Hamilton | | | | Hybrid | | | | Hybrid | | |  |
|  | | | | | | | | | | | | | | | | | | | | | |
| *DNA metrics* | | | | | | | | | | | | | | | | | | | | | |
| n | 71 | | | 8 | | | 8 | | | 8 | | | | 16 | | | | 31 | | |  |
|  | **Statistics** | **VV** | **CV** | **Statistics** | **VV** | **CV** | **Statistics** | **VV** | **CV** | **Statistics** | **VV** | **CV** | **Statistics** | | | **VV** | **CV** | **Statistics** | **VV** | **CV** | **KW P value** |
| DNA (ng/ul) (QT) (CV if >3.5) | 34.58±52.50  17.1 [3.11; 298] | 71 | 69 | 74.14±86.11  24.45 [9.40; 242] | 8 | 8 | 16.448±10.52  14.25 [5.4; 36.7] | 8 | 8 | 25.66±27.13  17.75 [4.4; 88.7] | 8 | 8 | 44.792±74.04  14.85 [3.11; 298] | | | 16 | 15 | 26.07±33.28  17.1 [3.4; 184] | 31 | 30 | 0.598 |
| A 260/280 (u) (QL) (CV if >2) | 2.02± 0.219  1.97 [1.50; 3.06] | 71 | 30 | 2.058± 0.093  2.045 [1.93; 2.21] | 8 | 5 | 2.186± 0.145  2.195 [1.97; 2.36] | 8 | 7 | 2.107± 0.393  1.965 [1.9; 3.06] | 8 | 3 | 1.921± 0.141  1.9 [1.5; 2.12] | | | 16 | 4 | 1.997± 0.209  1.95 [1.58; 2.6] | 31 | 11 | **0.004** |
| A 260/230 (u) (QL) (CV if >2) | 0.884±0.684  0.67 [0.1; 2.28] | 71 | 6 | 0.59± 0.448  0.38 [0.23; 1.49] | 8 | 0 | 0.303± 0.162  0.25 [0.18; 0.67] | 8 | 0 | 1.439± 0.909  2 [0.1; 2.28] | 8 | 2 | 1.183± 0.784  1.125 [0.26; 2.2] | | | 16 | 2 | 0.814± 0.539  0.75 [0.14; 2.12] | 31 | 2 | **0.005** |
| Delta Cq (u) (QL) (CV if <5) | 0.192±1.541  0.3 [-4.7; 3.6] | 71 | 71 | 0.838±0.851  0.45 [0.1; 2.5] | 8 | 8 | 0.437±0.722  0.15 [-0.4; 1.7] | 8 | 8 | -0.185±1.369  -0.5 [-1.6; 2] | 8 | 8 | -1.181±1.818  -0.7 [-4.7; 1.4] | | | 16 | 16 | 0.769±1.282  0.8 [-2.61; 3.6] | 31 | 31 | **0.001** |
| Quality Control post Fragmentation (bp) (QT) (CV if included between 150 and 300) | 235.1±35.73  232 [173; 315] | 71 | 69 | 197.9±17.59  207.5 [173; 216] | 8 | 8 | 197.1±13.23  195.5 [181; 212] | 8 | 8 | 220.8±25.49  219 [187; 265] | 8 | 8 | 226.8±28.62  228.5 [174; 272] | | | 16 | 16 | 262.4±26.21  261 [203; 315] | 31 | 29 | **<0.001** |
| Pre capture libraries metric (ng/ul) (QL) (CV if >20) | 47.43±7.298  48.7 [26.4; 60] | 71 | 71 | 46.64±3.613  47 [41.6; 52] | 8 | 8 | 49.85±1.790  49.65 [47.7; 53] | 8 | 8 | 30.7±3.001  30.45 [26.4; 35.6] | 8 | 8 | 49.71±3.373  49.25 [44.4; 57] | | | 16 | 16 | 50.16±5.089  50 [39.9; 60] | 31 | 31 | **<0.001** |
| Enriched libraries metric (ng/ul) (QL) (CV if >3) | 16.7±7.959  18.5 [1.53; 31.9] | 71 | 69 | 12.161±4.368  13.95 [5.58; 16.4] | 8 | 8 | 17.49±8.652  20.6 [3.4; 25.8] | 8 | 8 | 19.06±1.694  18.6 [17; 21.7] | 8 | 8 | 10.125±5.233  8.96 [3.34; 21.6] | | | 16 | 16 | 20.45±8.173  23 [1.53; 31.9] | 31 | 29 | **<0.001** |
| *QC Enriched libraries (bp) (CV if included between 250 and 300) | NA | 2 | NA | NE | 0 | 0 | NE | 0 | 0 | NE | 0 | 0 | NE | | | 0 | 0 | NA | 2 | NA | NE |
|  | | | | | | | | | | | | | | | | | | | | | |
| *RNA metrics* | | | | | | | | | | | | | | | | | | | | | |
| n | 59 | | | 6 | | | 6 | | | 8 | | | | 15 | | | | 24 | | |  |
|  | **Statistics** | **VV** | **CV** | **Statistics** | **VV** | **CV** | **Statistics** | **VV** | **CV** | **Statistics** | **VV** | **CV** | **Statistics** | | | **VV** | **CV** | **Statistics** | **VV** | **CV** | **KW P value** |
| RNA (ng/ul) (QT) (CV if >10.5) | 78.3±63.40  66 [12.3; 312] | 59 | 59 | 70.33±41.27  70.95 [23.7; 120] | 6 | 6 | 131.4±105.13  89.7 [30.1; 312] | 6 | 6 | 46.1±52.67  30.65 [12.3; 170] | 8 | 8 | 104.74±58.11  86 [36; 235.8] | | | 15 | 15 | 61.23±50.90  45.5 [13.1; 200.1] | 24 | 24 | **0.005** |
| RNA A260/280 (QL) (CV if >2) | 1.942±0.101  1.96 [1.6; 2.2] | 59 | 6 | 2±0.109  2 [1.9; 2.2] | 6 | 1 | 1.917±0.098  1.95 [1.8; 2] | 6 | 0 | 1.976±0.089  2 [1.76; 2.05] | 8 | 1 | 1.871±0.066  1.9 [1.7; 1.97] | | | 15 | 0 | 1.968±0.102  1.98 [1.6; 2.2] | 24 | 4 | **<0.001** |
| RNA A260/230 (QL) (CV if >2) | 1.123±0.635  1.36 [0.03; 1.95] | 59 | 0 | 1.105±0.563  1.21[0.09; 1.6] | 6 | 0 | 1.113±0.609  1.1 [0.17; 1.75] | 6 | 0 | 0.89±0.817  0.87 [0.03; 1.95] | 8 | 0 | 1.66±0.314  1.7 [0.6; 1.9] | | | 15 | 0 | 0.871±0.575  0.79 [0.16; 1.94] | 24 | 0 | **0.001** |
| DV200 (%) (QL) (CV if >20) | 59.2±16.71  63.7 [2.6; 86.9] | 59 | 58 | 59.33±15.49  64.7 [36.3; 73.2] | 6 | 6 | 60.32±17.52  56.6 [41.7; 83.5] | 6 | 6 | 61.29±18.73  68.1 [33.2; 82.5] | 8 | 8 | 65.25±10.79  67.2 [42.6; 77.8] | | | 15 | 15 | 54.4±18.92  55.95 [2.6; 86.9] | 24 | 23 | 0.455 |
| Pre capture libraries metric (ng/ul) (QL) (CV if >20) | 48.84±8.381  51 [25.7; 60] | 59 | 59 | 48.12±2.778  47.4 [45.4; 53] | 6 | 6 | 45.93±2.809  45.3 [43; 51] | 6 | 6 | 31.15±3.787  30.85 [25.7; 36.4] | 8 | 8 | 52.05±2.775  53 [46.1; 55] | | | 15 | 15 | 53.63±4.780  54.5 [39.7; 60] | 24 | 24 | **<0.001** |
| Enriched libraries metric (ng/ul) (QL) (CV if >3) | 7.033±4.702  6.51 [0.80; 19.6] | 59 | 45 | 5.145±3.821  5.755 [0.8; 9.03] | 6 | 4 | 9.685±6.081  7.45 [3.8; 18.6] | 6 | 6 | 6.893±6.023  5.615 [1.07; 17] | 8 | 4 | 6.981±5.636  5.25 [1.12; 19.6] | | | 15 | 10 | 6.920±3.372  6.925 [2.1; 13.8] | 24 | 21 | 0.635 |
| *QC Enriched libraries (bp) (CV if included between 250 and 300) | 267.7±17.133  262 [251; 300] | 7 (14) ** | 7 (14) ** | 269±9.899  269 [262; 276] | 2 | 2 | NE | All Post Hyb >3 | All Post Hyb >3 | 271.2±21.061  265 [255; 300] | 4 | 4 | 251 | | | 1 (5) | 1 (5) | NA | 0 (3) | NA | NE |

**Notes.** n: samples; LP: Library preparation; DNA: DeoxyriboNucleic Acid; RNA: RiboNucleic Acid; QT: variable of quantification; QL: variable of qualification; VV: Valid values (non-missing values); CV: compliant values in relation to the guideline threshold shown between parentheses; u: units; QC: Quality Control; NA: Not Available (i.e., QC not performed); NE: Not Expected; u: units; KW: Kruskal-Wallis test.

*Measured only for samples with enriched libraries metric ≤3. **QC performed only on 7 of out 14 samples reporting enriched libraries metric ≤3.

Data are expressed as mean±standard deviation and median [range] for numerical variables and absolute frequencies for the samples.

In **bold** the significant results (P<0.05), in *italic* the suggestive results (0.05<P<0.10).

**Supplementary Table S3.** Sequencing quality metrics

|  | **Overall** | | **Run 1** | **Run 2** | **Run 3** | **Run 4** | **Run 5** | **KW p-value** |
| --- | --- | --- | --- | --- | --- | --- | --- | --- |
| *DNA metrics* |  | **CV**  **(yes/no)** |  |  |  |  |  |  |
| n | 71 |  | 8 | 8 | 8 | 16 | 31 |  |
| Total number of reads passing filter (1,000,000u) | 142.60±54.92  120.71 [37.35; 252.80] | NE | 207.9±24.01  209.5 [180.3; 240.5] | 187.2± 53.59  197.4 [102.1; 244.3] | 226.6± 20.06  219.5 [202.8; 252.8] | 105.96± 15.41  107.80 [66.97; 125.81] | 111.50± 27.53  113.81 [37.35; 153.11] | **<0.001** |
| Median target coverage (u) | 648.1±324.13  684 [96; 1595] | NE | 1046±373.9  1174 [448; 1595] | 755.4±205.1  797.5 [434; 1086] | 818.5±228.6  804 [384; 1095] | 376.9±266.9  291 [96; 1074] | 613.7±247.7  628 [172; 1317] | **<0.001** |
| Chimeric reads (%) | 1.193±1.27  0.720 [0.05; 7.21] | NE | 1.519±1.480  0.880 [0.360; 4.870] | 1.855±1.424  1.470 [0.470; 4.070] | 0.916±0.625  0.670 [0.330; 2.090] | 1.008±0.900  0.700 [0.050; 2.930] | 1.104±1.456  0.610 [0.090; 7.21] | 0.259 |
| Exon 100x (%) | 95.76±8.56  98.50 [49.6; 99.50] | NE | 99.22±0.271  99.35 [98.7; 99.5] | 99.15±0.325  99.15 [98.6; 99.5] | 98.08±2.282  98.8 [92.5; 99.4] | 88.24±15.324  95.45 [49.6; 98.7] | 97.28±3.473  98.4 [81.8; 99.2] | **<0.001** |
| Read enrichment (%) | 68.79±8.869  70.50 [40.90; 82.8] | NE | 76.45±3.221  77.15 [70.5; 80.3] | 74.01±3.508  74.45 [68.2; 78.3] | 65.65±3.516  66.60 [60.3; 69.3] | 72.06±4.714  71.35 [64.8; 82.8] | 64.59±10.712  67.30 [40.9; 77.4] | **<0.001** |
| Reads that contain usable UMI information (%) | 99.89±0.035  99.9 [99.8; 99.9] | NE | 99.9±0  99.9 [99.9; 99.9] | 99.9±0  99.9 [99.9; 99.9] | 99.9±0  99.9 [99.9; 99.9] | 99.89±0.025  99.9 [99.8; 99.9] | 99.87±0.046  99.9 [99.8; 99.9] | NP |
| Mean target coverage (u) | 676.4±333.83  698.8 [110.4; 1619] | NE | 1069.2±384.16  1188.3 [457.6; 1619] | 784.6± 215.03  831.6 [446.1; 1124.5] | 873± 274.32  820.1 [403.8; 1261.4] | 413± 292.21  314.5 [110.4; 1182.3] | 632.4± 247  660.9 [185.1; 1382.9] | **<0.001** |
| Aligned reads (%) | 93.36±7.874  96.10 [55.40; 99.30] | NE | 97.11±2.741  97.85 [90.50; 98.80] | 97.50±1.452  98.05 [94.90; 99.10] | 94.34±3.708  96.45 [87.90; 97.20] | 86.23±13.200  90.15 [55.40; 98.20] | 94.76±3.793  96.00 [83.80; 99.30] | **<0.001** |
| Percent of foreign DNA estimated to be in the sample (%) | 3.73±4.595  2.40 [0.20; 25.70] | NE | 2.362±2.988  1.300 [0.200; 9.000] | 2.362±1.744  2.250 [0.300; 4.900] | 4.85±2.965  5.15 [0.30; 8.00] | 5.506±7.543  1.750 [0.400; 25.700] | 3.229±3.564  2.700 [0.200; 18.800] | 0.405 |
| Unique reads passing filter (%) | 100 ± 0 | NE | 100 ± 0 | 100 ± 0 | 100 ± 0 | 100 ± 0 | 100 ± 0 | NP |
| Target 04x mean (%) | 92.51 ± 6.052  94.20 [64.30; 96.90] | NE | 94.72 ± 1.377  95.40 [92.60; 96] | 96.17 ± 0.577  96.3 [94.9; 96.7] | 91.59 ± 11.088  95.45 [64.30; 96.8] | 88.25 ± 8.093  90.6 [67.10; 95.70] | 93.42 ± 2.804  94.10 [86.80; 96.90] | **<0.001** |
| Target 100x (%) | 95.09±9.026  98.10 [47.10; 99.40] | NE | 98.76±0.443  98.75 [97.90; 99.40] | 98.74±0.480  98.95 [97.70; 99.10] | 97.67±2.296  98.35 [92.10; 99.10] | 86.59±15.908  94.2 [47.10; 98.20] | 96.91±3.376  98.10 [82.40; 99.00] | **<0.001** |
| Target 250x (%) | 82.49±24.88  94.40 [0.40; 98.40] | NE | 96.70±2.294  97.20 [91.40; 98.40] | 96.22±2.761  97.50 [90.40; 98.20] | 93.38±7.127  96.55 [77.40; 98.20] | 56.36±33.249  61.85 [0.40; 96.30] | 85.96±19.09  94.40 [23.00; 97.30] | **<0.001** |
| Contamination score (u) (CV if ≤3106 or [>3106 and Contamination Pvalue≤0.049]) | 2494±3981.38  1362 [12; 30413] | 68 / 3 | 2441±2980.9  1433 [73; 7351] | 1763.2±1573.1  1305 [291; 5031] | 3192±2245.1  3320 [211; 6119] | 1527.1±2163.0  374 [72; 7416] | 3015.1±5455.9  2021 [12; 30413] | 0.367 |
| Contamination P value (u) (CV if ≤0.049 when contamination score >3106) | 0.584±0.451  0.966 [0.000; 1.000] | 15 / 3* | 0.646±0.435  0.833 [0.001; 1.000] | 0.601±0.422  0.726 [0.035; 1.000] | 0.295±0.440  0.063 [0.000; 1.000] | 0.692±0.470  1.000 [0.000; 1.000] | 0.582±0.450  0.966 [0.000; 1.000] | 0.486 |
| Coverage MAD (u) (CV if ≤0.210) | 0.164±0.047  0.161 [0.070; 0.321] | 61 / 10 | 0.156±0.045  0.167 [0.093; 0.211] | 0.144±0.035  0.156 [0.070; 0.182] | 0.157±0.020  0.163 [0.122; 0.179] | 0.194±0.060  0.174 [0.112; 0.321] | 0.157±0.043  0.161 [0.096; 0.259] | 0.330 |
| Median bin CNV target (u) (CV if ≥1) | 8.915±4.165  8.900 [1.500; 20.900] | 71 / 0 | 14.14±4.644  15.35 [6.80; 20.90] | 10.46±2.726  10.55 [5.80; 14.50] | 11.72±3.172  12.00 [5.50; 15.70] | 5.562±3.358  4.700 [1.500; 13.600] | 8.174±3.024  8.200 [2.500; 16.700] | **<0.001** |
| Median insert size (u) (CV if ≥70) | 110.3±16.45  113 [73; 149] | 71 / 0 | 108.4±7.327  110.5 [95; 117] | 107.1±7.059  106.0 [96; 116] | 110.8±6.713  114.5 [99; 116] | 94.62±20.848  88.00 [73; 149] | 119.5±12.50  120 [87; 139] | **<0.001** |
| Median exon coverage (u) (CV if ≥150) | 665.1±336.3  686 [99; 1646] | 68 / 3 | 1073.5±382.4  1183.5 [457; 1646] | 773.6±215.5  812.5 [435; 1118] | 860.8±258.0  830 [394; 1158] | 390.6±279.4  301.5 [99; 1137] | 622.8±254.2  652 [166; 1363] | **<0.001** |
| Exon 50x (%) (CV if ≥90) | 98.59±1.912  99.1 [87; 99.7] | 70 / 1 | 99.54±0.130  99.6 [99.3; 99.7] | 99.51±0.145  99.5 [99.3; 99.7] | 99.28±0.249  99.3 [98.8; 99.6] | 96.89±3.407  98.3 [87; 99.3] | 98.81±0.720  99 [96.2; 99.5] | **<0.001** |
| Unstable (MSI) sites (u) (CV if ≥40) | 103.1±36.86  122 [2; 125] | 64 / 7 | 120.8± 5.47  123.5 [109; 124] | 122.2±1.98  122.5 [119; 125] | 119.2±4.559  119 [112; 124] | 44.31±37.42  46.5 [2; 123] | 119.7±8.90  122 [89; 125] | **<0.001** |
|  |  |  |  |  |  |  |  |  |
| *RNA metrics* |  |  |  |  |  |  |  |  |
| n | 59 |  | 6 | 6 | 8 | 15 | 24 |  |
| Total number of reads passing filter (1,000,000u) | 25.57±6.331  29.049 [5.122; 30.006] | NE | 25.19±7.487  30 [14.3; 30] | 30±0.004  30 [29.99; 30.01] | 26.81±5.152  29.64 [16.91; 30] | 24.33±5.997  27.18 [12.25; 30] | 24.929±7.198  28.12 [5.122; 30] | 0.170 |
| Scaled median gene coverage (u) | 4592.4±1540.793  5186.7 [692.1; 6533.6] | NE | 4453±1690  5202 [2187; 6065] | 5835±306  5847 [5396; 6245] | 4554±1624  5139 [2280; 6452] | 4059±1615  4603 [1231; 6534] | 4662.9±1534  5203.2 [692.1; 6241.3] | 0.106 |
| Total reads on target region (1,000,000u) (CV≥9) | 23.261±6.243  26.094 [4.548; 28.974] | 57 / 2 | 23.34±7.734  27.97 [12.81; 28.97] | 28.00±0.428  28.13 [27.21; 28.42] | 23.11±6.220  26.23 [13.58; 28.76] | 22.45±6.013  24.51 [11.10; 28.94] | 22.616±6.689  24.766 [4.548; 28.099] | *0.086* |
| RNA median insert size (u) (CV≥80) | 101.2±13.78  102 [76; 148] | 58 / 1 | 108.33±15.56  112 [86; 123] | 110.7±14.12  109 [93; 133] | 99.62±17.17  104 [76; 124] | 95±10.82  96 [81; 112] | 101.5±12.62  101 [86; 148] | 0.127 |
| Chimeric reads (%) | 0.251±0.527  0.180 [0.070; 4.210] | NE | 0.175±0.052  0.160 [0.11; 0.26] | 0.191±0.027  0.190 [0.15; 0.23] | 0.676±1.427  0.175 [0.14; 4.21] | 0.198±0.051  0.2 [0.07; 0.29] | 0.176±0.073  0.17 [0.12; 0.51] | *0.090* |
| Target reads (%) | 90.56±5.492  92.50 [59.20; 96.60] | NE | 91.67±4.960  93.25 [82.70; 96.60] | 93.33±1.427  93.80 [90.70; 94.70] | 85.65±12.005  89.30 [59.20; 95.90] | 91.70±3.112  92.70 [84.70; 96.50] | 90.51±2.993  91.25 [83.20; 94.40] | 0.145 |
| Median gene coverage 500x (%) (CV if ≤ 93) | 60.71± 13.216  57.71 [42.95; 117.08] | 58 / 1 | 67.94±12.657  68.36 [50.47; 88.51] | 54.38±6.067  55.18 [45.81; 62.69] | 64.90±12.58  62.04 [46.15; 86.10] | 64.64±17.84  60.29 [46.54; 117.08] | 56.62±9.891  52.98 [42.95; 83.46] | *0.076* |
|  |  |  |  |  |  |  |  |  |
| *Run overall metrics* |  |  |  |  |  |  |  |  |
| PF_READS_perc (%) (CV if ≥55) | 85.86±2.765  86.2 [81.4, 89] | 5 / 0 | 86.0 | 81.4 | 89.0 | 86.2 | 86.7 | NE |
| Q30_R1 (%) (CV if ≥ 80) | 93.76±0.545  93.9 [92.9; 94.4] | 5 / 0 | 93.9 | 92.9 | 94.4 | 93.7 | 93.9 | NE |
| Q30_R2 (%) (CV if ≥ 80) | 93.08±1.413  93.5 [91.3; 94.8] | 5 / 0 | 92.0 | 91.3 | 94.8 | 93.5 | 93.8 | NE |

**Note.** n: samples; CV: compliant values in relation to the guidelines threshold shown between parentheses; u: units; DNA: DeoxyriboNucleic Acid; RNA: RiboNucleic Acid; NE: Not Expected;

UMI: Unique Molecular Identifiers; MAD: Median Absolute Deviation; MSI: MicroSatellite Instability; PF: Passing filter; Q30_R1: pecent of R1 reads with quality score > 30;

Q30_R2: pecent of R2 reads with quality score > 30; CNV: Copy Number Variation; KW: Kruskal-Wallis test; NP: Not performed because of poor or null variability.

Data are expressed as mean ± standard deviation and median [range] for numerical variables, and absolute frequencies for samples. In **bold** the significant results (P<0.05), in *italic* the suggestive results (0.05<P<0.10). *Among subjects with contamination score bigger than 3106. The contamination score is calculated to estimate foreign DNA contamination. It is based on VAF distribution of SNPs and it corresponds to the sum of all the log-likelihood scores across all positions.

**Supplementary Table S4.** Bioinformatics analysis workflow

| 1. FASTQ Generation |
| --- |
| 1. DNA analysis using the following methods |
| - DNA Alignment and Realignment |
| - Read Collapsing |
| - Indel Realignment and Read Stitching |
| - Small Variant Calling |
| - Small Variant Filtering |
| - Copy Number Variant Calling |
| - Phased Variant Calling |
| - Variant Merging |
| - Annotation |
| - Tumor Mutational Burden |
| - Microsatellite Instability Status |
| - Contamination Detection |
| 1. RNA analysis using the following methods |
| - Downsampling |
| - Read Trimming |
| - Alignment |
| - Duplicate Marking |
| - Fusion Calling |
| - RNA Fusion Filtering |
| - Splice Variant Calling |
| - Annotation |
| - Fusion Merging |
| 1. Quality Control |
| - Run QC |
| - DNA Sample QC |
| - RNA Sample QC |

**Supplementary** **Table S5.** Correlation analysis between median coverage (by depth) and wet metrics [1st run]

| 1^st^ run | **SNV** | | | | **CNV** | | | | **RNA** | | |
| --- | --- | --- | --- | --- | --- | --- | --- | --- | --- | --- | --- |
|  | Q2 | Q2 | Q2 | Q2 | Q2 | Q2 | Q2 | Q2 | Q2 | Q2 | Q2 |
|  | 50X | 100X | 250X | 500X | 50X | 100X | 250X | 500X | 5X | 10X | 50X |
| *DNA metrics (n=8)* |  |  |  |  |  |  |  |  |  |  |  |
| DNA | NP | NP | NP | τ=-0.367  P=0.249  95%CI=-0.767; 0.033 | NP | NP | NP | -0.214  0.513  -0.663; 0.235 | - | - | - |
| A 260/280 | NP | NP | NP | *0.576*  *0.070*  *-0.015; 1* | NP | NP | NP | 0.357  0.275  -0.205; 0.920 | - | - | - |
| A 260/230 | NP | NP | NP | -0.373  0.247  -0.784; 0.037 | NP | NP | NP | -0.218  0.510  -0.677; 0.240 | - | - | - |
| Delta Cq | NP | NP | NP | -0.053  0.868  -0.948; 0.842 | NP | NP | NP | -0.509  0.124  -1.000; 0.199 | - | - | - |
| Quality Control post Fragmentation | NP | NP | NP | 0.480  0.137  -0.220; 1 | NP | NP | NP | 0.218  0.510  -0.240; 0.677 | - | - | - |
| Pre-capture libraries metric | NP | NP | NP | -0.052  0.869  -0.529; 0.424 | NP | NP | NP | 0.071  0.827  -0.308; 0.451 | - | - | - |
| Enriched libraries metric | NP | NP | NP | *0.587*  *0.068*  *-0.001; 1* | NP | NP | NP | 0.363  0.272  -0.208; 0.936 | - | - | - |
|  |  |  |  |  |  |  |  |  |  |  |  |
| *RNA metrics (n=6)* |  |  |  |  |  |  |  |  |  |  |  |
| RNA | - | - | - | - | - | - | - | - | 0.276  0.444  -0.444; 0.996 | 0.2  0.573  -0.626; 1 | 0.467  0.189  -0.010; 0.943 |
| RNA A260/280 | - | - | - | - | - | - | - | - | *-0.644*  *0.094*  *-1; 0.435* | -0.544  0.150  -1; 0.079 | *-0.701*  *0.064*  *-1; 0.042* |
| RNA A260/230 | - | - | - | - | - | - | - | - | 0.357  0.330  -0.353; 1 | 0.414  0.251  -0.280; 1 | 0  1  -0.605; 0.604 |
| DV200 | - | - | - | - | - | - | - | - | **0.828**  **0.022**  **0.553; 1** | **0.733**  **0.038**  **0.432; 1** | 0.466  0.189  0.165; 0.768 |
| Pre-capture libraries metric | - | - | - | - | - | - | - | - | 0  1  -0.382; 0.382 | 0.066  0.851  -0.235; 0.368 | 0.067  0.851  -0.732; 0.865 |
| Enriched libraries metric | - | - | - | - | - | - | - | - | **0.828**  **0.021**  **0.553; 1** | **0.866**  **0.015**  **0.565; 1** | 0.333  0.348  -0.329; 0.996 |

**Note.** n: number of samples; Q2: median across-sample (patient) median percent of exon covered. RNA: RiboNucleic Acid; SNV: Single-Nucleotide Variant; CNV: Copy Number Variant; τ: Kendall’s Tau rank correlation coefficient; P: P value; 95%CI= 95% Confidence Interval; NP: Not performed because of poor or null variability; -: not computed. In **bold** the significant results (P<0.05), in *italic* the suggestive results (0.05<P<0.10).

**Supplementary** **Table S6.** Correlation analysis between median coverage (by depth) and wet metrics [2nd run]

| 2^nd^ run | **SNV** | | | | **CNV** | | | | **RNA** | | |
| --- | --- | --- | --- | --- | --- | --- | --- | --- | --- | --- | --- |
|  | Q2 | Q2 | Q2 | Q2 | Q2 | Q2 | Q2 | Q2 | Q2 | Q2 | Q2 |
|  | 50X | 100X | 250X | 500X | 50X | 100X | 250X | 500X | 5X | 10X | 50X |
| *DNA metrics (n=8)* |  |  |  |  |  |  |  |  |  |  |  |
| DNA | NP | NP | NP | τ=0.262  P=0.411  95%CI=-0.411; 0.935 | NP | NP | NP | 0.262  0.411  -0.411; 0.935 | - | - | - |
| A 260/280 | NP | NP | NP | -0.107  0.740  -0.829; 0.615 | NP | NP | NP | -0.106  0.741  -0.829; 0.616 | - | - | - |
| A 260/230 | NP | NP | NP | 0.262  0.411  -0.411; 0.935 | NP | NP | NP | 0.262  0.411  -0.411; 0.935 | - | - | - |
| Delta Cq | NP | NP | NP | 0.052  0.869  -0.826; 0.931 | NP | NP | NP | 0.052  0.869  -0.826; 0.931 | - | - | - |
| Quality Control post Fragmentation | NP | NP | NP | 0  1  -0.709; 0.709 | NP | NP | NP | 0  1  -0.709; 0.709 | - | - | - |
| Pre-capture libraries metric | NP | NP | NP | 0.053  0.868  -0.842; 0.948 | NP | NP | NP | 0.053  0.868  -0.842; 0.948 | - | - | - |
| Enriched libraries metric | NP | NP | NP | *0.587*  *0.068*  *-0.001;1* | NP | NP | NP | *0.587*  *0.068*  *-0.001;1* | - | - | - |
|  |  |  |  |  |  |  |  |  |  |  |  |
| *RNA metrics (n=6)* |  |  |  |  |  |  |  |  |  |  |  |
| RNA | - | - | - | - | - | - | - | - | -0.115  0.770  -0.631; 0.400 | -0.086  0.822  -0.784; 0.612 | 0.067  0.851  -0.813; 0.946 |
| RNA A260/280 | - | - | - | - | - | - | - | - | -0.404  0.343  -0.981; 0.172 | -0.603  0.142  -1; 0.285 | -0.545  0.150  -1; 0.025 |
| RNA A260/230 | - | - | - | - | - | - | - | - | -0.577  0.143  -1; 0.134 | **-0.774**  **0.042**  **-1; -0.336** | **-0.866**  **0.015**  **-1; -0.565** |
| DV200 | - | - | - | - | - | - | - | - | 0.577  0.143  -0.134; 1 | **0.774**  **0.042**  **0.336; 1** | **0.733**  **0.038**  **0.256; 1** |
| Pre-capture libraries metric | - | - | - | - | - | - | - | - | -0.115  0.770  -0.631; 0.400 | 0.086  0.821  -0.532; 0.705 | 0.466  0.189  -0.241; 1 |
| Enriched libraries metric | - | - | - | - | - | - | - | - | 0.577  0.143  -0.134; 1 | 0.086  0.821  -0.885; 1 | -0.2  0.573  -1; 0.705 |

**Note.** n: number of samples; Q2: median across-sample (patient) median percent of exon covered. RNA: RiboNucleic Acid; SNV: Single-Nucleotide Variant; CNV: Copy Number Variant; τ: Kendall’s Tau rank correlation coefficient; P: P value; 95%CI= 95% Confidence Interval; NP: Not performed because of poor or null variability; -: not computed. In **bold** the significant results (P<0.05), in *italic* the suggestive results (0.05<P<0.10).

**Supplementary** **Table S7.** Correlation analysis between median coverage (by depth) and wet metrics [3^rd^ run]

| 3^rd^ run | **SNV** | | | | **CNV** | | | | **RNA** | | |
| --- | --- | --- | --- | --- | --- | --- | --- | --- | --- | --- | --- |
|  | Q2 | Q2 | Q2 | Q2 | Q2 | Q2 | Q2 | Q2 | Q2 | Q2 | Q2 |
|  | 50X | 100X | 250X | 500X | 50X | 100X | 250X | 500X | 5X | 10X | 50X |
| *DNA metrics (n=8)* |  |  |  |  |  |  |  |  |  |  |  |
| DNA | NP | NP | NP | *τ=0.576*  *P=0.070*  *95%CI=-0.515; 1* | NP | NP | NP | *0.576*  *0.070*  *-0.515; 1* | - | - | - |
| A 260/280 | NP | NP | NP | **-0.721**  **0.028**  **-1; -0.220** | NP | NP | NP | **-0.721**  **0.028**  **-1; -0.220** | - | - | - |
| A 260/230 | NP | NP | NP | **0.721**  **0.028**  **0.220; 1** | NP | NP | NP | **0.721**  **0.028**  **0.220; 1** | - | - | - |
| Delta Cq | NP | NP | NP | 0.262  0.411  -0.247; 0.771 | NP | NP | NP | 0.262  0.411  -0.247; 0.771 | - | - | - |
| Quality Control post Fragmentation | NP | NP | NP | 0.052  0.869  -0.506; 0.611 | NP | NP | NP | 0.052  0.869  -0.506; 0.611 | - | - | - |
| Pre-capture libraries metric | NP | NP | NP | -0.053  0.868  -0.948; 0.842 | NP | NP | NP | -0.053  0.868  -0.948; 0.842 | - | - | - |
| Enriched libraries metric | NP | NP | NP | 0.157  0.621  -0.614; 0.928 | NP | NP | NP | 0.157  0.621  -0.614; 0.928 | - | - | - |
|  |  |  |  |  |  |  |  |  |  |  |  |
| *RNA metrics (n=8)* |  |  |  |  |  |  |  |  |  |  |  |
| RNA | - | - | - | - | - | - | - | - | *0.491*  *0.098*  *-0.473; 1* | 0.357  0.216  -0.074; 0.789 | 0.214  0.458  -0.260; 0.689 |
| RNA A260/280 | - | - | - | - | - | - | - | - | -0.055  0.866  -0.161; 0.050 | 0.157  0.622  -0.117; 0.431 | 0.157  0.622  -0.337; 0.651 |
| RNA A260/230 | - | - | - | - | - | - | - | - | 0.416  0.161  0.013; 0.819 | 0.286  0.322  -0.199; 0.771 | 0  1  -0.560; 0.560 |
| DV200 | - | - | - | - | - | - | - | - | 0.340  0.252  -0.013; 0.692 | 0.071  0.804  -0.403; 0.546 | -0.071  0.804  -0.400; 0.257 |
| Pre-capture libraries metric | - | - | - | - | - | - | - | - | 0.038  0.898  -0.474; 0.550 | 0  1  -0.523; 0.523 | -0.286  0.322  -0.728; 0.157 |
| Enriched libraries metric | - | - | - | - | - | - | - | - | *0.567*  *0.056*  *-0.408; 1* | 0.286  0.322  -0.340; 0.912 | 0.143  0.621  -0.381; 0.667 |

**Note.** n: number of samples; Q2: median across-sample (patient) median percent of exon covered. RNA: RiboNucleic Acid; SNV: Single-Nucleotide Variant; CNV: Copy Number Variant; τ: Kendall’s Tau rank correlation coefficient; P: P value; 95%CI= 95% Confidence Interval; NP: Not performed because of poor or null variability; -: not computed. In **bold** the significant results (P<0.05), in *italic* the suggestive results (0.05<P<0.10).

**Supplementary** **Table S8.** Correlation analysis between median coverage (by depth) and wet metrics [4^th^ run]

| 4^th^ run | **SNV** | | | | **CNV** | | | | **RNA** | | |
| --- | --- | --- | --- | --- | --- | --- | --- | --- | --- | --- | --- |
|  | Q2 | Q2 | Q2 | Q2 | Q2 | Q2 | Q2 | Q2 | Q2 | Q2 | Q2 |
|  | 50X | 100X | 250X | 500X | 50X | 100X | 250X | 500X | 5X | 10X | 50X |
| *DNA metrics (n=16)* |  |  |  |  |  |  |  |  |  |  |  |
| DNA | NP | τ=-0.012  P=0.952  95%CI=-0.525; 0.500 | 0.165  0.399  -0.242; 0.574 | **0.449**  **0.035**  **0.113; 0.784** | NP | -0.012  0.952  -0.525; 0.500 | 0.114  0.567  -0.308; 0.536 | 0.280  0.173  -0.111; 0.673 | - | - | - |
| A 260/280 | NP | -0.092  0.667  -0.607; 0.421 | -0.215  0.289  -0.648; 0.216 | -0.327  0.138  -0.656; 0.001 | NP | -0.092  0.667  -0.607; 0.421 | -0.222  0.281  -0.663; 0.218 | -0.310  0.146  -0.674; 0.052 | - | - | - |
| A 260/230 | NP | 0.012  0.952  -0.467; 0.492 | 0.298  0.132  -0.112; 0.709 | **0.429**  **0.046**  **0.112; 0.745** | NP | 0.012  0.952  -0.467; 0.492 | 0.288  0.151  -0.115; 0.693 | *0.372*  *0.073*  *-0.337; 1* | - | - | - |
| Delta Cq | NP | 0.151  0.472  -0.300; 0.603 | -0.167  0.397  -0.584; 0.248 | -0.312  0.148  -0.666; 0.042 | NP | 0.151  0.472  -0.301; 0.604 | -0.115  0.566  -0.533; 0.302 | -0.262  0.207  -0.631; 0.106 | - | - | - |
| Quality Control post Fragmentation | NP | *0.397*  *0.056*  *-0.054; 0.849* | 0.082  0.673  -0.345; 0.510 | 0.012  0.952  -0.315; 0.341 | NP | *0.397*  *0.056*  *-0.054; 0.849* | 0.028  0.886  -0.407; 0.463 | -0.150  0.463  -0.551; 0.249 | - | - | - |
| Pre-capture libraries metric | NP | -0.187  0.369  -0.642; 0.266 | -0.323  0.101  -0.695; 0.047 | **-0.451**  **0.035**  **-0.773; -0.128** | NP | -0.187  0.369  -0.642; 0.266 | *-0.372*  *0.062*  *-0.754; 0.010* | *-0.368*  *0.074*  *-1; 0.365* | - | - | - |
| Enriched libraries metric | NP | **0.621**  **0.002**  **0.260; 0.981** | **0.743**  **<0.001**  **0.633; 0.852** | **0.575**  **0.007**  **0.256; 0.893** | NP | **0.621**  **0.002**  **0.260; 0.981** | **0.747**  **<0.001**  **0.643; 0.851** | **0.710**  **<0.001**  **0.516; 0.903** | - | - | - |
|  |  |  |  |  |  |  |  |  |  |  |  |
| *RNA metrics (n=15)* |  |  |  |  |  |  |  |  |  |  |  |
| RNA | - | - | - | - | - | - | - | - | -0.093  0.672  -0.359; 0.171 | 0.109  0.617  -0.259; 0.478 | 0  1  -0.330; 0.330 |
| RNA A260/280 | - | - | - | - | - | - | - | - | -0.153  0.543  -0.403; 0.097 | -0.382  0.123  -0.960; 0.197 | -0.328  0.134  -0.968; 0.312 |
| RNA A260/230 | - | - | - | - | - | - | - | - | 0.186  0.434  -0.378; 0.749 | 0.309  0.186  -0.191; 0.809 | 0.117  0.573  -0.361; 0.594 |
| DV200 | - | - | - | - | - | - | - | - | 0.207  0.352  -0.216; 0.629 | 0.109  0.617  -0.301; 0.520 | -0.039  0.842  -0.380; 0.302 |
| Pre-capture libraries metric | - | - | - | - | - | - | - | - | 0.019  0.932  -0.585; 0.624 | 0  1  -0.523; 0.524 | 0.040  0.841  -0.402; 0.482 |
| Enriched libraries metric | - | - | - | - | - | - | - | - | **0.453**  **0.042**  **0.022; 0.928** | **0.533**  **0.015**  **0.138; 0.929** | **0.650**  **<0.001**  **0.361; 0.940** |

**Note.** n: number of samples; Q2: median across-sample (patient) median percent of exon covered. RNA: RiboNucleic Acid; SNV: Single-Nucleotide Variant; CNV: Copy Number Variant; τ: Kendall’s Tau rank correlation coefficient; P: P value; 95%CI= 95% Confidence Interval; NP: Not performed because of poor or null variability; -: not computed. In **bold** the significant results (P<0.05), in *italic* the suggestive results (0.05<P<0.10).

**Supplementary Table S9.** Correlation analysis between median coverage (by depth) and wet metrics [5^th^ run]

| 5^th^ run | **SNV** | | | | **CNV** | | | | **RNA** | | |
| --- | --- | --- | --- | --- | --- | --- | --- | --- | --- | --- | --- |
|  | Q2 | Q2 | Q2 | Q2 | Q2 | Q2 | Q2 | Q2 | Q2 | Q2 | Q2 |
|  | 50X | 100X | 250X | 500X | 50X | 100X | 250X | 500X | 5X | 10X | 50X |
| *DNA metrics (n=31)* |  |  |  |  |  |  |  |  |  |  |  |
| DNA | NP | NP | τ=0.110  P=0.454  95%CI=-0.228; 0.448 | 0.152  0.267  -0.145; 0.450 | NP | NP | 0.017  0.906  -0.317; 0.352 | 0.178  0.191  -0.114; 0.472 | - | - | - |
| A 260/280 | NP | NP | -0.180  0.227  -0.403; 0.042 | -0.168  0.228  -0.422; 0.084 | NP | NP | -0.137  0.360  -0.361; 0.086 | -0.162  0.243  -0.421; 0.097 | - | - | - |
| A 260/230 | NP | NP | 0.023  0.872  -0.193; 0.240 | 0.094  0.489  -0.164; 0.354 | NP | NP | -0.035  0.814  -0.243; 0.173 | 0.111  0.414  -0.135; 0.358 | - | - | - |
| Delta Cq | NP | NP | *-0.246*  *0.097*  *-0.539; 0.046* | *-0.258*  *0.063*  *-0.519; 0.003* | NP | NP | -0.159  0.289  -0.451; 0.133 | *-0.259*  *0.061*  *-0.523; 0.005* | - | - | - |
| Quality Control post Fragmentation | NP | NP | -0.024  0.872  -0.298; 0.251 | -0.113  0.412  -0.391; 0.165 | NP | NP | 0.009  0.953  -0.276; 0.294 | -0.100  0.467  -0.364; 0.165 | - | - | - |
| Pre-capture libraries metric | NP | NP | -0.207  0.163  -0.420; 0.005 | -0.117  0.401  -0.358; 0.124 | NP | NP | -0.123  0.409  -0.328; 0.079 | -0.129  0.352  -0.375; 0.117 | - | - | - |
| Enriched libraries metric | NP | NP | **0.376**  **0.010**  **0.086; 0.665** | **0.332**  **0.015**  **0.063; 0.601** | NP | NP | **0.443**  **0.003**  **0.145; 0.740** | **0.342**  **0.012**  **0.072; 0.612** | - | - | - |
|  |  |  |  |  |  |  |  |  |  |  |  |
| *RNA metrics (n=24)* |  |  |  |  |  |  |  |  |  |  |  |
| RNA | - | - | - | - | - | - | - | - | -0.057  0.704  -0.384; 0.269 | -0.030  0.841  -0.309; 0.249 | -0.026  0.862  -0.262; 0.212 |
| RNA A260/280 | - | - | - | - | - | - | - | - | -0.084  0.591  -0.390; 0.222 | -0.105  0.494  -0.418; 0.207 | -0.129  0.393  -0.432; 0.176 |
| RNA A260/230 | - | - | - | - | - | - | - | - | -0.026  0.859  -0.370; 0.313 | 0.007  0.960  -0.337; 0.352 | -0.032  0.823  -0.343; 0.278 |
| DV200 | - | - | - | - | - | - | - | - | 0.126  0.403  -0.184; 0.437 | 0.105  0.483  -0.197; 0.406 | 0.061  0.673  -0.209; 0.332 |
| Pre-capture libraries metric | - | - | - | - | - | - | - | - | 0.252  0.103  -0.020; 0.525 | 0.207  0.174  -0.073; 0.488 | 0.194  0.194  -0.110; 0.499 |
| Enriched libraries metric | - | - | - | - | - | - | - | - | **0.340**  **0.024**  **0.086; 0.595** | 0.239  0.109  -0.038; 0.516 | 0.149  0.308  -0.168; 0.467 |

**Note.** n: number of samples; Q2: median across-sample (patient) median percent of exon covered. RNA: RiboNucleic Acid; SNV: Single-Nucleotide Variant; CNV: Copy Number Variant; τ: Kendall’s Tau rank correlation coefficient; P: P value; 95%CI= 95% Confidence Interval; NP: Not performed because of poor or null variability; -: not computed. In **bold** the significant results (P<0.05), in *italic* the suggestive results (0.05<P<0.10).

**Supplementary Table S10.** DNA and RNA sequential phases of the post-sequencing

| **DNA Phases:** |
| --- |
| 1. Alignment and Realignment |
| 1. Reads Collapsing |
| 1. Indel Realignment and Read Stitching |
| 1. Small Variant Calling |
| 1. Small Variant Filtering |
| 1. Copy Number Variant Calling |
| 1. Phased Variant Calling |
| 1. Variant Merging |
| 1. Annotation |
| 1. Tumor Mutational Burden |
| 1. Microsatellite Instability Status |
| 1. Contamination Detection |
|  |
| **RNA Phases:** |
| 1. Downsampling |
| 1. Read Trimming |
| 1. Alignment |
| 1. Duplicate Marking |
| 1. Fusion Calling |
| 1. RNA Fusion Filtering |
| 1. Splice Variant Calling |
| 1. Annotation |
| 1. Fusion Merging |

**Supplementary** **Table S11.** Sequencing quality control metrics by sample and feature type

| **Feature** | **Sample Type** | **Feature type** |
| --- | --- | --- |
| Total number of reads passing filter (u): Absolute number of reads which passed the quality filter | DNA | reads quality |
| Read enrichment (%): Percentage of reads enriched for the target regions | DNA | reads quality |
| Aligned reads (%): Percentage of aligned reads | DNA | reads quality |
| Unique reads passing filter (%): Percentage of unique reads which passed the quality filter | DNA | reads quality |
| Median target coverage (u): Median fragment coverage of target bases | DNA | coverage |
| Exon 100x (%): Percentage of Exons mapped at 100x | DNA | coverage |
| Mean target coverage (u): Mean fragment coverage of target bases | DNA | coverage |
| Target 04x mean (%): Mean of percentage of reads on target with 04x of coverage | DNA | coverage |
| Target 100x (%): Percentage of reads on target with 100x of coverage | DNA | coverage |
| Target 250x (%): Percentage of reads on target with 250x of coverage | DNA | coverage |
| Chimeric reads (%): Percentage of reads with half position mapping in a different genome location | DNA | unusual reads |
| Percent of foreign DNA estimated to be in the sample (%): Percentage of foreign DNA to estimate a sample contamination | DNA | unusual reads |
| Reads that contain usable UMI information (%): Percentage reads with the Unique Molecular Identifier | DNA | UMI |
| Total number of reads passing filter (u): Absolute number of reads which passed the quality filter | RNA | reads quality |
| Scaled median gene coverage (u): The median of median gene coverage scaled by gene length. Median coverage is calculated for each gene, and then scaled by the respective gene’s length, and finally divided by the total length of all targeted genes | RNA | coverage |
| Target reads (%): Percentage of reads on target region including partially coverage reads map | RNA | coverage |
| Median gene coverage 500x (%): Median of percentage of genes with coverage above 500x | RNA | coverage |
| Chimeric reads (%): Percentage of reads with half position mapping in a different genome location | RNA | unusual reads |

**Note.** UMI: Unique Molecular Identifiers

**Supplementary** **Table S12.** Descriptive statistics of coverage

|  | **Overall** | | **Run 1** | **Run 2** | **Run 3** | **Run 4** | **Run 5** | **KW P value**  **(on runs)** |
| --- | --- | --- | --- | --- | --- | --- | --- | --- |
| LP (Manual/Hamilton/Hybrid) |  | | Manual | Manual | Hamilton | Hybrid | Hybrid |  |
|  |  | CV  (yes/no) |  |  |  |  |  |  |
| *SNV Q2 (%)* |  |  |  |  |  |  |  |  |
| Number of exons  (533 per sample) | 37843 |  | 4264 | 4264 | 4264 | 8528 | 16523 |  |
| n | 71 |  | 8 | 8 | 8 | 16 | 31 |  |
| Depth 50X (CV if ≥75) | 100±0  100 [100; 100] | 71 / 0 | 100±0  100 [100; 100] | 100±0  100 [100; 100] | 100±0  100 [100; 100] | 100±0  100 [100; 100] | 100±0  100 [100; 100] | NP |
| Depth 100X (CV if ≥75) | 99.12±4.806  100 [62.36; 100] | 70 / 1 | 100±0  100 [100; 100] | 100±0  100 [100; 100] | 100±0  100 [100; 100] | 96.11±9.763  100 [62.36; 100] | 100±0  100 [100; 100] | NP |
| Depth 250X (CV if ≥75) | 88.71± 28.48  100 [0; 100] | 61 / 10 | 100± 0  100 [100; 100] | 100±0  100 [100; 100] | 100±0  100 [100; 100] | 66.02±42.97  88.21 [0; 100] | 91.69±24.17  100 [0; 100] | NP |
| Depth 500X (CV if ≥75) | 64.01 ±44.93  100 [0; 100] | 44 / 27 | 96.77 ±8.271  100 [76.39; 100] | 86.66 ±27.323  100 [24.79; 100] | 86.11 ±35.010  100 [0; 100] | 18.85 ±40.262  0 [0; 100] | 67.32 ±42.112  91.84 [0; 100] | **<0.001** |
| **KW (on depth)**: P value= NP |  |  |  |  |  |  |  |  |
|  |  |  |  |  |  |  |  |  |
| *CNV Q2 (%)* |  |  |  |  |  |  |  |  |
| Number of exons  (412 per sample) | 29252 |  | 3296 | 3296 | 3296 | 6592 | 12772 |  |
| n | 71 |  | 8 | 8 | 8 | 16 | 31 |  |
| Depth 50X (CV if ≥75) | 100±0  100 [100; 100] | 71 / 0 | 100±0  100 [100; 100] | 100±0  100 [100; 100] | 100±0  100 [100; 100] | 100±0  100 [100; 100] | 100±0  100 [100; 100] | NP |
| Depth 100X (CV if ≥75) | 99.12±4.004  100 [62.36; 100] | 70 / 1 | 100±0  100 [100; 100] | 100±0  100 [100; 100] | 100±0  100 [100; 100] | 96.65±8.097  100 [69.40; 100] | 100±0  100 [100; 100] | NP |
| Depth 250X (CV if ≥75) | 90 ± 26.82  100 [0; 100] | 62 / 9 | 100± 0  100 [100; 100] | 100±0  100 [100; 100] | 100±0  100 [100; 100] | 66.77±42.94  88.98 [0; 100] | 94.25±19.25  100 [0; 100] | NP |
| Depth 500X (CV if ≥75) | 66.92±43.51  100 [0; 100] | 45 / 26 | 97.59 ±6.818  100 [80.72; 100] | 90.16 ±20.481  100 [43.14; 100] | 86.73 ±35.110  100 [0; 100] | 21.77 ±39.703  0 [0; 100] | 71.21 ±40.347  99.24 [0; 100] | **<0.001** |
| **KW (on depth)**: P value= NP |  |  |  |  |  |  |  |  |
|  |  |  |  |  |  |  |  |  |
| *RNA Q2 (%)* |  |  |  |  |  |  |  |  |
| Number of exons  (336 per sample) | 19824 |  | 2016 | 2016 | 2688 | 5040 | 8064 |  |
| n | 59 |  | 6 | 6 | 8 | 15 | 24 |  |
| Depth 5X (CV if ≥75) | 94.17 ±17.62  100 [0; 100] | 56 / 3 | 81.34±29.35  93.48 [24.63; 100] | 99.55±1.113  100 [97.27; 100] | 86.58±35.006  98.98 [0; 100] | 96.23±14.400  100 [44.18; 100] | 97.28±3.701  98.68 [88.25; 100] | **0.012** |
| Depth 10X (CV if ≥75) | 93.32 ± 17.89  98.98 [0.00; 100] | 55 / 4 | 79.87 ± 29.59  93.1 [24.63; 100] | 99.39 ± 1.177  100 [97.06; 100] | 85.96 ± 34.75  98.49 [0; 100] | 96.16 ± 14.38  100 [44.18; 100] | 95.84 ± 5.959  98.01 [76.09; 100] | **0.004** |
| Depth 50X (CV if ≥75) | 81.07± 30.47  96.96 [0; 100] | 46 / 13 | 61.35±44.60  80.51 [0; 97.21] | 97.14±1.744  97.83 [94.04; 98.63] | 75.05±35.409  92.56 [0; 100] | 93.58±19.379  98.36 [23.63; 100] | 76.18±31.463  93.47 [0; 99.83] | **<0.001** |
| **KW (on depth)**: P value <**0.001** |  |  |  |  |  |  |  |  |
